# Phytobiotic Essential Oils as Antibiotic Alternatives in Aquaculture: Antimicrobial and Antioxidant Properties of Garlic, Thyme, Thyme Conehead, Rosemary, and Eucalyptus

**DOI:** 10.1002/fsn3.71739

**Published:** 2026-04-24

**Authors:** Calinoiu Lavinia Florina, Plamada Diana, Teleky Bernadette‐Emoke, Stefanescu Bianca‐Eugenia, Chrysanthos Stergiopoulos, Magdalini Krokida, Ioannis Maramathas, Papadaki Sofia, Moustogianni Anna, Panagiota Anagnostopoulou, Vodnar Dan Cristian

**Affiliations:** ^1^ Department of Food Science, Faculty of Food Science and Technology University of Agricultural Sciences and Veterinary Medicine of Cluj‐Napoca Cluj‐Napoca Romania; ^2^ Institute of Life Sciences University of Agricultural Sciences and Veterinary Medicine of Cluj‐Napoca Cluj‐Napoca Romania; ^3^ National Technical University of Athens, School of Chemical Engineering, Laboratory of Process Analysis and Design Zografou Athens Greece; ^4^ Dignity Private Company Zografou Athens Greece; ^5^ Zoonomi S.A. Perigiali Korinthias Greece

**Keywords:** antimicrobial, antioxidant, aquaculture, encapsulation, essential oils, ionic gelation, spray drying

## Abstract

The increasing threat of antimicrobial resistance in aquaculture demands sustainable alternatives to conventional antibiotics. Plant‐derived essential oils are attractive candidates owing to their antimicrobial, antioxidant, and immunomodulatory activities, yet their volatility and chemical instability hinder incorporation into aquafeeds. This study integrated chemical profiling, bioactivity assessment, and spray‐drying microencapsulation to evaluate five Eos, garlic, thyme, conehead thyme, rosemary, and eucalyptus, as functional feed additives (illustrated in the Graphical abstract below). Gas chromatography–mass spectrometry (GC–MS) revealed distinct chemotypes: garlic oil was rich in propyl propane thiosulfonate (30.3%); thyme oil in thymol (35.1%) and p‐cymene (31.6%); conehead thyme oil in carvacrol (19.9%); rosemary oil in eucalyptol (43.0%); and eucalyptus oil in eucalyptol (68.9%). Antioxidant capacity varied markedly, with conehead thyme (34.7 μmol/mL) and garlic (17.8 μmol/mL) outperforming thyme (12.7 μmol/mL), while rosemary (0.79 μmol/mL) and eucalyptus (0.32 μmol/mL) were weak. Antimicrobial testing showed potent activity for garlic and both thyme oils against *Escherichia coli* , *Pseudomonas aeruginosa* , *Staphylococcus aureus* , and others, with several minimum inhibitory concentrations comparable to or lower than gentamicin. All EOs exhibited antifungal activity, in some cases approaching or exceeding fluconazole. Spray‐drying with maltodextrin or starch produced spherical microcapsules (mean 19–28 μm) with encapsulation efficiencies of ~67%–71% and process yields of 53%–79%. Release in PBS (pH 7.4) was rapid, consistent with porous particle morphology. Overall, spray‐dried EO microcapsules preserved bioactivity and handling stability, supporting their feasibility as natural antibiotic alternatives in aquaculture feeds and motivating in vivo validation.

## Graphical abstract

Microencapsulation of garlic, thyme, rosemary, and eucalyptus essential oils using maltodextrin and starch matrices enhances their stability, bioactivity, and controlled release, supporting their use as natural antimicrobial and antioxidant alternatives to antibiotics in aquaculture.

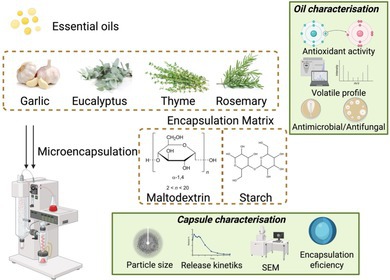

## 1 Introduction

The rapid expansion of aquaculture has been accompanied by extensive use of antibiotics to prevent and treat infectious diseases. Although effective in the short term, the misuse of antibiotics has accelerated the development of antimicrobial resistance, contributed to environmental contamination, and raised concerns regarding food safety (Angane et al. 2022). These challenges highlight the urgent need for sustainable alternatives that can safeguard fish health without promoting resistance. The global natural feed additives market is projected to grow from ~USD 9.0 billion in 2025 to ~USD 19.3 billion by 2035 (CAGR ≈7.9%) (Pinto and Santos 2017).

Plant‐derived essential oils (EOs) constitute a complex mixture of volatile metabolites, primarily monoterpenes, phenolic monoterpenes, and occasional organosulfur compounds that exert broad antimicrobial and antioxidant effects (Reis et al. 2022). Their antimicrobial activity is mediated through multiple simultaneous mechanisms, including perturbation of cytoplasmic membrane integrity, efflux of intracellular constituents, dissipation of transmembrane proton motive force, and the generation of oxidative stress within microbial cells. Similarly, their antioxidant action derives from radical scavenging, hydrogen‐atom transfer, and metal chelation by phenolic moieties. Such broad‐spectrum bioactivity renders EOs promising candidates to replace synthetic antimicrobials and antioxidants in food, pharmaceutical, and feed applications (Yammine et al. 2023; Sousa et al. 2022).

Among the investigated oils, garlic ( *Allium sativum* ) oil (GO) is rich in organosulfur compounds such as allicin, diallyl disulfide, and diallyl trisulfide, which display strong antimicrobial, antifungal, and antioxidant activity by disrupting enzymes, damaging microbial membranes, and modulating oxidative stress. These attributes support its use in aquaculture to enhance disease resistance and reduce dependence on antibiotics (El‐Saber Batiha et al. 2020; Pashaki et al. 2020; Alexopoulos et al. 2011). Thyme ( *Thymus vulgaris* ) oil (TO), dominated by thymol and carvacrol, exhibits pronounced antibacterial and antifungal activity through membrane permeabilization and ROS generation, while also boosting fish immune responses in feed applications (Burt 2004; Hyldgaard et al. 2012; Dawood et al. 2022).

Conehead thyme ( *Thymus capitatus* ) oil (TCO), with a particularly high carvacrol content and secondary thymol, shows even stronger antimicrobial and antioxidant efficacy, conferring preservation potential and functional benefits in aquaculture nutrition (Salehi et al. 2018; Brenes and Roura 2010; Nazzaro et al. 2017). In contrast, terpene‐rich oils such as rosemary (*Salvia rosmarinus*) oil (RO) and eucalyptus ( *Eucalyptus globulus* ) oil (EUO) display distinct but complementary activity profiles. RO, composed mainly of 1,8‐cineole, α‐pinene, camphor, and borneol, demonstrates notable antioxidant capacity that stabilizes lipids in feeds and provides moderate antimicrobial action (Nieto et al. 2018; Jan et al. 2017; Yang et al. 2023). EUO, dominated by 1,8‐cineole (up to ~70%) together with limonene and α‐pinene, exerts antimicrobial, antifungal, and anti‐inflammatory effects, though typically weaker than phenolic‐rich oils, while contributing respiratory and immunomodulatory benefits (Cermelli et al. 2008; Silva et al. 2003; Khalil et al. 2024). Taken together, phenolic‐rich oils (garlic, thyme, conehead thyme) provide strong antimicrobial potency, whereas terpene‐rich oils (rosemary, eucalyptus) offer pronounced antioxidant and modulatory functions, suggesting their combined application may provide a synergistic strategy for sustainable aquaculture. The volatility of EOs, poor water solubility, and susceptibility to thermal and oxidative degradation lead to significant losses during processing, handling, and in vivo applications. These physicochemical limitations undermine retention, reproducibility, and biological availability in applied systems. Encapsulation strategies, designed to entrap oils within protective carriers, are increasingly employed to address these challenges. By stabilizing the oil and controlling release, encapsulation can preserve bioactivity and enhance functionality in various matrices (Zhao et al. 2023; Phanse et al. 2022).

The rapid expansion of aquaculture has been accompanied by extensive use of antibiotics to prevent and treat infectious diseases. Although antibiotics remain effective in the short term, their overuse has accelerated the emergence of antimicrobial resistance genes (AMRg), contributed to environmental pollution, and raised significant food safety concerns. These issues underscore the urgent need for sustainable alternatives capable of preserving fish health without fostering resistance. Plant‐derived EOs represent promising candidates for this purpose. Rich in terpenes (α‐pinene, camphor), phenolics (thymol and carvacrol), and sulfur‐containing compounds (allicin, diallyl disulfide), they exhibit broad‐spectrum antimicrobial, antioxidant, and immunomodulatory activities (Mohammed et al. 2025). Key bioactives, such as allicin (garlic), thymol and carvacrol (thyme), and eucalyptol (rosemary and eucalyptus), are known to inhibit major aquaculture pathogens like *Aeromonas hydrophila* (Nya and Austin 2009). However, the practical application of EOs in aquafeeds is limited by their volatility, low water solubility, and instability during processing and gastrointestinal digestion. Encapsulation technologies provide a potential solution by entrapping bioactive compounds within protective matrices, thereby enhancing their stability, bioavailability, and enabling controlled release (Zabot et al. 2022). Techniques such as spray drying, electrospraying, ionic gelation, and emulsification followed by freeze‐drying have been widely applied to food and pharmaceutical compounds but remain underexplored in aquaculture applications (Zhu et al. 2021; Yousefi et al. 2023). This study investigates the chemical composition, antimicrobial and antioxidant activities, and encapsulation performance of EOs from garlic, thyme, conehead thyme, rosemary, and eucalyptus. By integrating bioactivity assessment with encapsulation outcomes, the study aims to evaluate the feasibility of these EOs as functional feed additives and natural alternatives to antibiotics in aquaculture.

To date, various encapsulation techniques have been validated in food, pharmaceutical, and nutraceutical sectors. Among encapsulation methods, spray drying has emerged as the most widely applied approach due to its scalability, cost‐effectiveness, and suitability for feed production, although the high processing temperatures may partially degrade thermolabile compounds (Mohammed et al. 2020). Comparative reviews show that carrier type, solid concentration, drying or crosslinking conditions, and particle morphology all influence encapsulation yield, retention stability, and release kinetics (Anal and Singh 2007).

Recent reports further underscore the functional advantages of encapsulated oils: nanoencapsulation improves stability, modulates controlled release, and enhances antimicrobial efficacy compared to free oils, thus supporting their deployment in challenging matrices (Fakhariha et al. 2025).

This study investigates the chemical composition, antimicrobial and antioxidant properties, and spray‐drying encapsulation performance of EOs derived from garlic, thyme, conehead thyme, rosemary, and eucalyptus. By integrating bioactivity profiling with encapsulation outcomes, the work aims to evaluate the feasibility of these EOs as functional feed additives and natural alternatives to antibiotics in aquaculture.

## 2 Materials and Methods

### 2.1 Materials and Chemicals

The essential oils were sourced from a certified Greek producer, Chiron Kentavros, located in Pelion, Thessaly, Greece. A variety of carrier matrices were employed to optimize encapsulation efficiency and stability. As encapsulation matrix, the following carriers were employed: maltodextrin, zein, whey protein isolate (WPI) acquired from Sigma‐Aldrich (Steinheim, Germany), chitosan by Merck (Darmstadt, Germany), and Arabic gum (Art. No. 51198). The WPI contents are 82% protein, 7.5% fat, 4% sugar, and 6% water. For the antimicrobial and antioxidant assays, the chemicals used, including Mueller–Hinton broth and agar, peptone, tryptic soy broth, and resazurin, were obtained from BioMérieux (Marcy l'Étoile, France). Further chemicals needed for the preparation of digestion were of analytical grade and double distilled water was used to prepare all solutions and emulsions. For the HPLC analyses, NaH_2_PO_4_ was supplied by Merck (Darmstadt, Germany), and purified water was prepared using a Direct‐Q UV system from Millipore (St. Louis, MO, USA). Both HPLC (purity 99.9% HPLC) and GC–MS standards were obtained by BioMerieux (Marcy l'Etoile, France) or Sigma‐Aldrich (Steinheim, Germany).

### 2.2 Methods

#### 2.2.1 Volatile Profile Characterization

EOs were diluted to 0.2 mg/mL in hexane before analysis. Gas chromatography–mass spectrometry (GC–MS) was performed on a GC‐2030 system coupled to a GC–MS‐QP2020 NX single quadrupole mass detector (Shimadzu Corporation, Kyoto, Japan), equipped with an SP‐2340 fused silica capillary column (30 m × 0.25 mm i.d., 0.20 μm film thickness; Supelco, Bellefonte, PA, USA). The oven program was set as follows: initial temperature, 50°C; ramped to 100°C at 10°C min^−1^; further increased to 220°C at 15°C min^−1^; and held at 220°C for 7 min. Helium served as the carrier gas at a constant flow of 1 mL min^−1^. The injector temperature was maintained at 220°C with a split ratio of 20:1. Mass spectra were acquired over the mass‐to‐charge ratio (m/z) range of 40–400. Compound identification was achieved by comparing the obtained spectra with those in the NIST and Wiley mass spectral libraries. Quantification was performed using calibration curves prepared from commercially available pure reference standards, all of which exhibited coefficients of determination (*r* ^2^) ≥ 0.99. Distinct chemical profiles were observed among rosemary, thyme, conehead thyme, garlic, and eucalyptus oils, reflecting variability that directly contributes to their differential biological activities (Vasileiou et al. 2025).

#### 2.2.2 Antioxidant Activity

The antioxidant capacity of the extracts was determined using the DPPH radical scavenging assay and expressed as Trolox equivalent antioxidant capacity (TEAC) in μmol/mL (Diaconeasa et al. 2015; Baliyan et al. 2022). A DPPH solution (25 mg/L in methanol) was prepared, and 3.9 mL of this solution was mixed with 0.1 mL of extract. After incubation in the dark for 30 min at room temperature, absorbance was measured at 515 nm using a Bel Photonics M51 UV–Vis spectrophotometer (Bel Engineering s.r.l., Monza, Italy). Antioxidant capacity was calculated from the reduction in absorbance relative to a control (methanol instead of the extract) using a Trolox calibration curve, with all standard curves exhibiting coefficients of determination (*r* ^2^) greater than 0.99. All the results were expressed as Trolox equivalent antioxidant capacity (TEAC) in μmol/mL.

#### 2.2.3 Antimicrobial and Antifungal Assays

##### 2.2.3.1 Culture Condition and Inocullum Preparation

To determine the minimum inhibitory concentration (MIC), the following bacterial strains were evaluated: *Staphylococcus aureus* ATCC 29213, *Escherichia coli* ATCC 25922, *Salmonella enterica* ATCC 14028, *Pseudomonas aeruginosa* ATCC 27853, *Staphylococcus epidermidis* ATCC 12228, and *Listeria monocytogenes* DSMZ 115292. Each strain was initially cultured in 10 mL of an appropriate sterile broth medium: Tryptic Soy Broth (TSB) for *E. coli* , *S. aureus* , and *P. aeruginosa* ; Nutrient Broth (NB) for *S. epidermidis* ; and Tryptic Soy Yeast Extract (TSYE) for *L. monocytogenes* . Cultures were activated by incubating the broth tubes at 37°C for 24 h. The purity of each inoculum was verified via microscopic examination of Gram‐stained smears. Following incubation, the spread plate technique was used to inoculate agar plates with each microorganism from its respective broth culture, ensuring adequate distribution and colony isolation. The bacterial morphology was subsequently confirmed through optical microscopy. To prepare for MIC testing, multiple colonies from each agar plate were transferred into a sterile saline solution (8.5 g/L NaCl), and the suspension was adjusted to achieve the turbidity of a 0.5 McFarland standard using a spectrophotometer at a 600 nm wavelength, corresponding to approximately 1.5 × 10^8^ CFU/mL.

To obtain the working inoculum concentration used in the MIC assay (1.5 × 10^5^ CFU/mL), serial decimal dilutions were performed in sterile broth medium (two sequential 1:100 dilutions followed by a 1:10 dilution). The final bacterial concentration was periodically verified by the standard plate count method (spread plating on agar followed by colony enumeration after incubation) and expressed as CFU/mL. The selected inoculum density is consistent with standard broth microdilution protocols and was chosen to ensure reproducible growth kinetics and reliable resazurin colorimetric endpoints while minimizing inoculum‐dependent variability.

For assessing antifungal activity, the following fungal strains were utilized: *Aspergillus awamori* DSMZ 63272, *Aspergillus niger* ATCC 6275, *Rhizopus oligosporus* ATCC 22959, *Aspergillus brasiliensis* ATCC 16404, *Aspergillus terreus* DSMZ 23081, and *Actinomucor elegans* ATCC 22963. All strains were cultured on Potato Dextrose Agar (PDA) as the growth medium. To prepare the inoculum, a spore suspension at a concentration of 10^6^ spores/mL was made by harvesting spores from dormant agar plates. For inoculation on PDA plates, 100 μL of the prepared spore suspension was evenly spread on the solidified agar surface using a sterile Drigalsky spatula to ensure uniform distribution. The spores were detached from the agar plates using a 0.1% Tween 80 solution and sterile glass beads to facilitate dispersion. The inoculated plates were then incubated at 30°C for 5 days, allowing a consistent layer of black spores to develop across the agar surface.

##### 2.2.3.2 Minimum Inhibitory Activity

The MIC was determined using a resazurin‐based microtiter plate antibacterial/antifungal assay. In each well of a 96‐well microtiter plate, 100 μL of sterile medium (specific to each microorganism) and 100 μL of the sample were added. Twelve serial 2‐fold dilutions were performed across the wells of each row by transferring 100 μL from well to well, discarding 100 μL from the final well. To each well, 10 μL of inoculum was added, with a final bacterial concentration of 1.5 × 10^5^ CFU/mL (prepared by serial dilution from a 0.5 McFarland suspension as described in Section 2.2.3.1) and 10^6^ CFU/mL for fungal and yeast strains. Gentamicin (0.4 mg/mL in water) served as the positive control (C+) for bacteria, while fluconazole (1.5 mg/mL in DMSO) was used as the positive control (C+) for fungi. The water served as the negative control (C–) for all samples. The plates were incubated at 37°C for bacteria (20–22 h) and at 30°C for fungi and yeasts (20–48 h). After incubation, 20 μL of filter‐sterilized resazurin aqueous solution (0.2 mg/mL) was added to each well, followed by an additional 2‐h incubation period. MIC values were recorded as the lowest concentration at which no color change from blue to pink occurred, indicating complete inhibition of bacterial or fungal growth (Appendix S1).

### 2.3 Encapsulation of Essential Oils Using Spray Drying

A Buchi Mini Spray Dryer B‐290 (Switzerland) equipped with an outlet filter was used for spray drying. All ingredients were weighed on a scale (KERN PLS6200‐2A, Germany) before addition. All samples were prepared by mixing maltodextrin with all the ingredients in a 1:1 (v/v) ratio in 100 mL of distilled water in a 500 mL bottle for 10 min. They were then homogenized for 1 min at 15,000 rm. to ensure a homogenous mix with minimal particle size (Kinematica Polytron PT 6100 D, Switzerland). Drying parameters were optimized to preserve the physicochemical integrity of the EO microcapsules while ensuring efficient moisture removal. The inlet temperature of the drying chamber was set to 121°C, with the atomizing air flow rate maintained at 100% of the unit's nominal capacity. Atomization was achieved using a standard two‐fluid nozzle with an orifice diameter of 0.3 mm, producing fine droplets for rapid and uniform drying.

The outlet (secondary cooling) chamber temperature was continuously monitored and remained within the range of 40 –50 C, mitigating potential thermal degradation of thermolabile constituents. The flow rate was set to 500 mL/h, and the pumping rate of the product mixture was set to 26% for all samples (Lipan et al. 2020).

## 3 Encapsulation Efficiency, Particle Properties, and Release Kinetics

### 3.1 Encapsulation Efficiency, Yield, and Morphology

Encapsulation efficiency (EE%) was assessed by quantifying the amount of bioactive retained within the microparticles compared to the total amount initially added. The encapsulated fractions were extracted with hexane and quantified by GC–MS, while the total oil content was determined after complete dissolution of microparticles in ethanol. EE% was calculated as:

(1) $$\text{Encapsulation efficiency}\;\left(\%\right)=\frac{\mathrm{Me}}{\mathrm{Mt}}\times 100$$

where Me is the amount of bioactive encapsulated and Mt is the total amount of bioactive initially used in the formulation.

Microencapsulation yield (Y%) was calculated according to:

(2) $$Y\left(\%\right)=\frac{\mathrm{Mr}}{\mathrm{Ms}}\times \;100$$

where Mr is the mass of recovered microparticles and Ms is the total mass of solids (wall material + bioactive) used in the feed formulation.

Particle size distribution was measured using a Laser Diffraction Particle Size Analyzer (Microtrac S3000/S3500 Series, Microtrac Inc., Montgomeryville, PA, USA). Results are reported as particle size ranges, midpoints, and mean values ± standard deviation (SD) based on triplicate measurements. Morphological characteristics were evaluated using Scanning Electron Microscopy (SEM, Quanta 200 FE, FEI Company, Hillsboro, OR, USA) at 5000× magnification. Before imaging, powders were mounted on aluminum stubs using double‐sided carbon tape, sputter‐coated with a thin gold layer under high‐vacuum conditions, and then observed under the same conditions.

### 3.2 In Vitro Release Kinetics

Release studies were performed in phosphate‐buffered saline (PBS, pH 7.4) at 25°C under gentle agitation. Spray‐dried microparticles were dispersed in the release medium at an initial concentration corresponding to 50 mg of microparticles per 50 mL PBS, containing the encapsulated essential oils. At predetermined time intervals (0, 5, 10, 20, 30, 40, and 50 h), aliquots were withdrawn, filtered through 0.45 μm membranes, and analyzed by GC–MS The withdrawn volume was replaced with fresh PBS to maintain sink conditions. Cumulative release (%) was calculated relative to the initial bioactive load. The time‐dependent releasevalues are presented in Table S1.

### 3.3 Statistical Analyses

All experiments were performed in triplicate, and results are expressed as mean ± standard deviation (SD). Data normality was assessed with the Shapiro–Wilk test (*p* > 0.05 indicating normal distribution). For comparisons among oils, one‐way ANOVA and post hoc Tukey tests were applied. Statistical analyses were conducted with GraphPad Prism version 9.3.0 (GraphPad Software Inc., San Diego, CA, USA). Pearson's correlation analysis was used to evaluate the relationships between microencapsulation yield, oil content, and processing temperature.

## 4 Results and Discussion

### 4.1 Volatile Profile of Essential Oils

**FIGURE 1:**
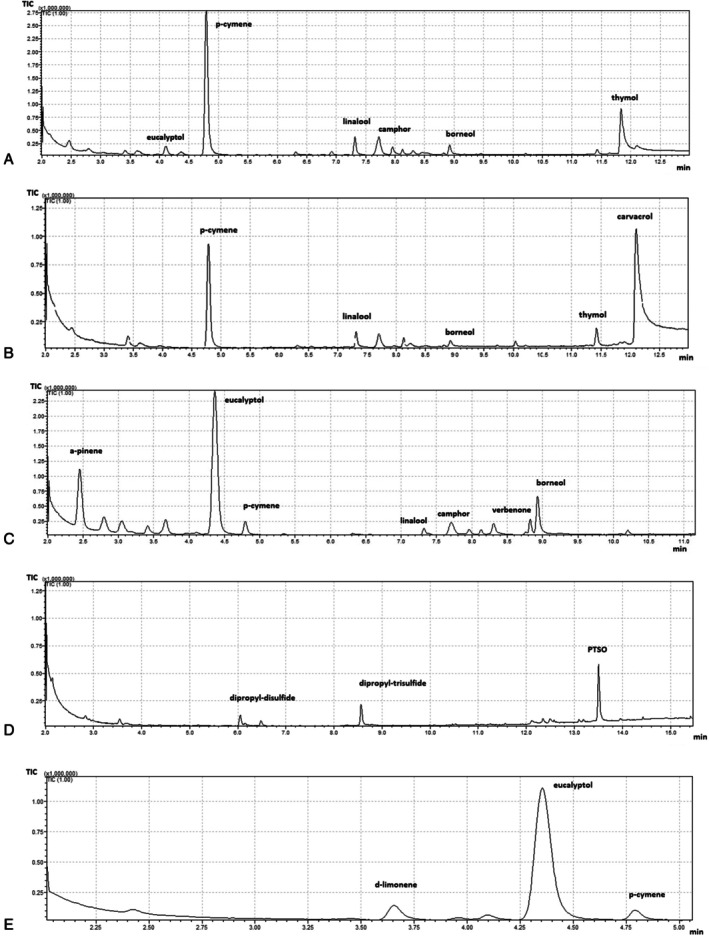
Representative GC–MS chromatogram with major peaks labeled of (A). thyme, (B). conehead thyme, (C). rosemary, (D). garlic, (E). eucalyptus EOs.

GC–MS analysis revealed distinct chemical profiles for the tested EOs (Table 1). Rosemary EO was primarily composed of eucalyptol (43.00% ± 8.87%), followed by α‐pinene (9.06% ± 1.98%), camphor (3.85% ± 0.10%), and borneol (3.57% ± 0.11%). Smaller amounts of *p*‐cymene (1.38% ± 0.05%), verbenone (1.24% ± 0.03%), and linalool (2.26% ± 0.08%) were also detected, as can be observed in Figure 1. This composition is consistent with cineole–camphor chemotypes of RO previously reported, where eucalyptol typically ranges from 20%–50% and α‐pinene, camphor, and borneol are major secondary constituents (al‐Sereiti et al. 1999). Variations in relative abundances are often attributed to geographical origin, harvest season, and extraction conditions (Santoyo et al. 2005). TO was dominated by thymol (35.1% ± 5.03%) and *p*‐cymene (31.6% ± 0.64%). Linalool (2.65% ± 0.08%) and borneol (2.50% ± 0.09%) were present at lower concentrations, along with trace levels of camphor and carvacrol. This profile matches the thymol chemotype, one of the major thyme oil variants, where thymol typically constitutes 20%–55% and p‐cymene 15%–35% (Satyal et al. 2016; Rota et al. 2008). The predominance of thymol, a phenolic monoterpene, is strongly associated with potent antibacterial and antifungal activities. TCO showed a different profile, with carvacrol (19.98% ± 0.79%) as the principal constituent, followed by p‐cymene (4.83% ± 1.39%) and linalool (2.22% ± 0.08%).

**TABLE 1:** Chemical composition of essential oils by GC–MS, expressed as % w/w (mean ± SD, *n* = 3), based on identification and quantification using pure reference standards.

Compounds	% w/w	SD
Rosemary essential oil		
Eucalyptol (1,8‐Cineol)	43.00	8.87
Pinene‐a	9.06	1.98
Camphor	3.85	0.18
Borneol	3.57	0.11
Linalool	2.26	0.08
Cymene‐p	1.38	0.05
Verbenone	1.24	0.03
Thyme essential oil		
Thymol	35.1	5.03
Cymene‐p	31.6	0.64
Linalool	2.65	0.08
Borneol	2.50	0.09
Camphor	2.01	0.01
Carvacrol	0.87	0.08
Eucalyptol (1,8‐Cineol)	0.13	0.03
Thyme conehead essential oil		
Carvacrol	19.98	0.79
Cymene‐p	4.83	1.39
Linalool	2.22	0.08
Borneol	0.20	0.02
Garlic essential oil		
Propyl propane thiosulfonate (PTSO)	30.3	4.34
Dipropyl trisulfide	1.72	0.07
Dipropyl disulfide	0.64	0.10
Eucalyptus essential oil		
Eucalyptol (1,8‐Cineol)	68.9	0.27
Limonene‐D	5.25	0.88
Cymene‐p	1.01	0.07

GO was characterized by sulfur‐derived compounds, mainly propyl propane thiosulfonate (PTSO) (30.3% ± 4.34%), dipropyl trisulfide (1.72% ± 0.07%), and dipropyl disulfide (0.64% ± 0.10%). Sulfur compounds such as PTSO and diallyl sulfides are characteristic of garlic oils and extracts, contributing to their broad‐spectrum antimicrobial activity (Iciek et al. 2009; Martins et al. 2016). The relatively high abundance of PTSO in this sample highlights its role as a potent antimicrobial agent through thiol–disulfide exchange and inhibition of key metabolic enzymes (e.g., alcohol dehydrogenase). EUO contained the highest proportion of eucalyptol (68.9% ± 0.27%), along with limonene‐D (5.25% ± 0.88%) and p‐cymene (1.01% ± 0.07%). This composition corresponds to the cineole chemotype widely described for *E. globulus* , where eucalyptol typically represents 60%–85% of the oil (Dhakad et al. 2018; Salem et al. 2018). Minor monoterpenes such as limonene and p‐cymene occur at low levels and may contribute synergistically to antimicrobial activity.

Comparative analysis reveals that terpene‐rich oils, such as those derived from rosemary and eucalyptus, are primarily composed of eucalyptol and other monoterpenes (Borges et al. 2019; Barbosa et al. 2016). In contrast, phenolic‐rich oils ( *T. vulgaris* and *T. capitatus* ) contain thymol and carvacrol as key bioactive compounds with potent antimicrobial properties. In comparison, GO was distinguished by its sulfur‐containing compounds, which confer broad‐spectrum antibacterial potential. These differences underpin the varied antimicrobial and antioxidant capacities observed across the tested oils. Encapsulation studies from the literature confirm that such compositional diversity also influences EE. A broad survey of spray‐dried EOs reported efficiencies ranging from as low as 9% for ginger oil to nearly 100% for citronella oil, depending largely on the wall material used (e.g., maltodextrin, gum Arabic, whey protein) and oil chemistry (Pudziuvelyte et al. 2025; Altay et al. 2024). In contrast, an experimental study using hemp protein isolate–gallic acid (HPI–GA) conjugates as encapsulating agents reported EE values between 40% and 88%, with oregano oil achieving the highest efficiency due to the amphiphilic properties of carvacrol, which favor droplet stabilization and retention (Zhang et al. 2025). These comparisons highlight that both the chemical nature of essential oils and the physicochemical properties of the encapsulating matrix critically determine encapsulation efficiency, stability, and release performance (Phanse and Chandra 2024). Phenolic‐rich oils such as thyme and conehead thyme generally display higher compatibility with protein‐ or polysaccharide‐based carriers, while terpene‐ and sulfur‐rich oils show more variable encapsulation outcomes depending on the formulation approach.

### 4.2 Antioxidant Activity

**FIGURE 2:**
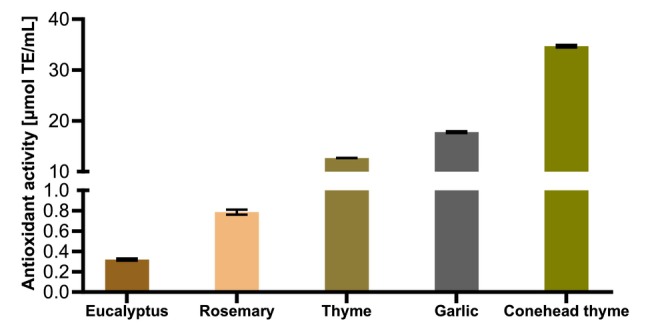
Antioxidant activity of essential oils [μmol TE/mL]. Results are expressed as mean ± SD (*n* = 3). Tukey's multiple comparison test was applied, with *p*‐values reported when > 0.05. Significance levels are indicated as ***p < 0.001.

Marked differences (*p* < 0.001) were observed among the tested oils (Figure 2). TCO exhibited the highest antioxidant activity, with a TEAC value of 34.7 ± 0.22 μmol/mL, indicating a strong capacity to neutralize free radicals. GO also demonstrated notable antioxidant potential, with a TEAC value of 17.78 ± 0.08 μmol/mL, ranking second among the tested oils. TO exhibited moderate activity (12.7 ± 0.06 μmol/mL), confirming the contribution of phenolic compounds, such as thymol and *p*‐cymene, to its antioxidant effect. In contrast, RO exhibited only weak antioxidant activity (0.794 ± 0.02 μmol/mL), despite its relatively high content of monoterpenes such as eucalyptol and α‐pinene. The lowest activity was recorded for eucalyptus essential oil (0.319 ± 0.01 μmol/mL), consistent with its composition, which is dominated by eucalyptol, a compound with limited antioxidant potential compared to phenolic constituents. Overall, oils rich in phenolic compounds (e.g., thymol and carvacrol in *T. vulgaris* and *T. capitatus* ) showed markedly stronger antioxidant properties than those dominated by monoterpenes or sulfur‐based compounds.

These results align with broader literature showing that phenolic‐rich EOs consistently outperform terpene‐dominated ones in antioxidant assays. For instance, Wang et al. (2010) reported that clove bud, cinnamon leaf, and thyme red essential oils, dominated by eugenol and thymol, had exceptionally low EC50 values (10–18 μg/mL) in ABTS radical scavenging assays, confirming the high efficacy of phenolic monoterpenes (Wang et al. 2010). Similarly, Chen et al. (2023) found that cinnamon, clove, and thyme oils exhibited the strongest antioxidant activities in both chemical (DPPH) and biological systems (fish oil emulsions and red blood cells), with activity correlated to eugenol and thymol content (Chen et al. 2023). In contrast, peppermint and lavender oils, rich in menthol and linalool, showed much weaker effects, consistent with our findings for rosemary and eucalyptus oils.

### 4.3 Antimicrobial and Antifungal Activity

All EOs demonstrated antimicrobial activity. Garlic and both thyme variants showed higher inhibition than gentamicin against *E. coli* , *P. aeruginosa* , and *S. aureus* . Conehead thyme and garlic EO were the most effective (Table 2). Antifungal testing confirmed broad inhibition, particularly for garlic and thyme oils (Table 3).

**TABLE 2:** Antimicrobial of essential oils.

Sample	Initial conc. (μL/mL)	Strain					
		Gram negative bacteria (−)		Gram positive bacteria (+)			
		*E. coli*	*S. enterica*	*P. aeruginosa*	*L. monocytogenes*	*S. epidermidis*	*S. aureus*
		Minimum inhibitory concentration (μL/mL)					
Eucalyptus	12.500	12.500	12.500	6.250	1.563	0.094	12.500
Garlic	0.781	1.563	1.563	0.391	0.195	0.014	0.781
Thyme	1.563	0.781	0.781	0.391	0.195	0.023	1.563
Thyme conehead	0.781	0.391	0.391	0.391	0.098	0.014	0.781
Rosemary	25.000	12.500	0.000	1.563	1.563	0.125	25.000
(C‐) SS + Tween 80 + EtOH (1:1:8)	n.b.	n.b.	12.500	12.500	12.500	n.b.	n.b.
(C+) Gentamicin 0.4 mg/mL	6.250	3.125	0.391	1.563	0.098	0.006	6.250

Abbreviations: EtOH, Ethanol; n.b., no bioactivity; SS, saline solution.

**TABLE 3:** Antifungal activity of essential oils.

Sample	Initial concentration (μL/mL)	Fungal strain						
		Gram‐negative bacteria (−)		Gram‐positive bacteria (+)				
		*C. parapsilosis*	*A. terreus*	*A. elegans*	*R. oligosporus*	*A. niger*	*A. awamori*	*A. brasiliensis*
		Minimum inhibitory concentration (μL/mL)						
Eucalyptus	100	—	3.125	—	1.563	0.098	—	—
Garlic	100	0.781	0.781	6.250	0.195	0.024	0.007	0.014
Thyme	100	0.391	1.563	3.125	0.391	0.098	0.063	0.063
Thyme conehead	100	0.098	6.250	0.781	0.781	0.195	0.078	0.078
Rosemary	100	25.000	n.b.	n.b.	n.b.	n.b.	2.000	0.500
(C‐) Saline solution + Tween 80 + EtOH (1:1:8)	100	n.b.	n.b.	n.b.	n.b.	n.b.	n.b.	n.b.
(C+) Fluconazol 1.5 mg/ml	400	187.500	187.500	n.b.	n.b.	n.b.	0.375	0.375

Abbreviations: EtOH, Ethanol; n.b., no bioactivity.

The antibacterial activity of the tested EOs was evaluated against both Gram‐negative and Gram‐positive bacteria (Table 2). Distinct differences in potency were observed, reflecting the oils' chemical compositions. GO exerted broad‐spectrum activity, with MICs of 1.563 μL/mL against *E. coli* and *S. enterica* , 0.391 μL/mL against *P. aeruginosa* , 0.195 μL/mL against *L. monocytogenes* , 0.014 μL/mL against *S. epidermidis* , and 0.781 μL/mL against *S. aureus* . The high efficacy is given by sulfur‐containing compounds, such as PTSO, which disrupt thiol‐dependent metabolic processes in bacteria (Cabello‐Gomez et al. 2022). This finding is consistent with previous studies, which have demonstrated that PTSO exhibits potent antibacterial and antiparasitic effects in fish (Cabello‐Gomez et al. 2022). However, the present study extends these observations by directly comparing the antimicrobial performance of several phytobiotic essential oils under identical experimental conditions and linking their activity to detailed GC–MS chemical profiles, providing a clearer evaluation of their relative potential as natural antibiotic alternatives in aquaculture.

TCO showed pronounced activity, especially against Gram‐positive species. The concentrations were 0.781 μL/mL for *S. aureus* , 0.014 μL/mL for *S. epidermidis* , and 0.391 μL/mL for *E. coli* , *S. enterica* , *P. aeruginosa* , and *L. monocytogenes* . These results align with the well‐established efficacy of carvacrol and thymol, which exert membranolytic and quorum‐sensing inhibitory actions (Alexopoulos et al. 2011). TO was also highly effective, with MICs ranging from 1.563 μL/mL ( *S. aureus* ) to 0.781 μL/mL ( *E. coli* ). This activity is attributed to thymol and p‐cymene content; these components synergize to increase membrane fluidity and permeability (Romulo et al. 2024).

EUO, dominated by eucalyptol, showed moderate efficacy: 12.500 μL/mL against *E. coli* , *S. enterica* , and *S. aureus* , 6.250 μL/mL against *P. aeruginosa* , 1.563 μL/mL against *L. monocytogenes* , and 0.094 μL/mL against *S. epidermidis* . These results reflect the generally weaker antibacterial potency of eucalyptol‐rich oils compared to phenolic‐rich oils (Kolygas et al. 2025).

RO was the least effective: with MICs of 12.500 μL/mL against *E. coli* , no detectable inhibition against *S. enterica* , 1.563 μL/mL against *P. aeruginosa* and *L. monocytogenes* , 0.125 μL/mL against *S. epidermidis* , and 25.000 μL/mL against *S. aureus* . Its poor performance is consistent with a chemical profile dominated by monoterpenes such as eucalyptol and α‐pinene, which have limited antibacterial action (Kolygas et al. 2025).

Relative to the positive control gentamicin, which inhibited strains at MICs ranging from 0.006 μL/mL ( *S. epidermidis* ) to 3.125 μL/mL ( *E. coli* ), garlic and thyme oils displayed comparable or superior efficacy against several strains, particularly against Gram‐positive pathogens. This highlights their potential as alternative or supplementary antimicrobial agents in aquaculture.

The antifungal activity of the tested EOs was quantitatively assessed by determining MICs against relevant fungi (Table 3). The results demonstrated marked differences in both spectrum and potency, which reflected the distinct chemical compositions of the oils. GO exhibited the most potent and broad‐spectrum activity, with MICs as low as 0.007 μL/mL (*A. awamori*), 0.014 μL/mL ( *A. brasiliensis* ), and 0.024 μL/mL ( *A. niger* ), up to 6.250 μL/mL for *A. elegans* . These results support the evidence from Pereira et al. (2021), who demonstrated potent inhibition of *Candida albicans* clinical isolates by garlic EO, thereby highlighting its role as a powerful antifungal agent (Pereira et al. 2021). CTO oil also displayed remarkable antifungal effects, with MICs of 0.098 μL/mL against *C. parapsilosis* , 0.781 μL/mL facing *A. elegans* and *R. oligosporus*, and 0.195 μL/mL toward *A. niger* . The high efficacy is attributed to carvacrol, the dominant phenolic monoterpene, which is known to disrupt ergosterol biosynthesis and compromise membrane integrity (Zhang et al. 2019).

TO EOs also showed high antimicrobial activity with MIC values of 0.391 μL/mL for *C. parapsilosis* and *A. niger* , 0.098 μL/mL for *A. awamori*, 0.063 μL/mL for *A. brasiliensis* , and 3.125 μL/mL for *A. elegans* . The antifungal effects are attributed to thymol and *p*‐cymene, whose synergistic interactions enhance cell membrane disruption and oxidative stress responses (Duan et al. 2024). EUO exhibited selective antifungal activity, with MICs of 3.125, 1.563, and 0.098 μL/mL against *Aspergillus terreus*, *Rhizopus oligosporus*, and *Aspergillus niger* respectively. No inhibition was recorded against *Candida parapsilosis* , *A. elegans* , *A. awamori*, or *A. brasiliensis* . This narrow spectrum is consistent with the dominance of eucalyptol in its composition, a monoterpene oxide known to exhibit weaker antifungal action compared to phenolic compounds such as thymol or carvacrol (Wang et al. 2019; Barboucha et al. 2024). RO was the least effective, showing inhibition only at 25.000 μL/mL for *C. parapsilosis* , 0.500 μL/mL for *A. brasiliensis* , and 2.000 μL/mL for *A. awamori*, with no measurable activity against other fungi. These findings suggest that RO has limited efficacy against the fungal strains examined in this study (Bozin et al. 2007). For example, RO glycolic extract inhibited the growth of *C. albicans* (for 72 h) in a *Galleria mellonella* survival model (Meccatti et al. 2022). Yuan et al. 2024 conducted a study utilizing the same matrix of RO encapsulated against the fungi *Colletotrichum gloeosporioides* with an MIC of 15.625 μL/mL, and fungicidal effect of 31.25 μL/mL (Yuan et al. 2024). RO's major chemical constituents, such as camphor, 1,8‐cineole, α‐pinene, and borneol, have also been implicated in disrupting fungal membranes and interfering with ergosterol biosynthesis (Shahina et al. 2022; Hendel et al. 2024).

Garlic and thyme oils, particularly conehead thyme, showed the highest antifungal potency, with MICs below 1 μL/mL for several strains and markedly surpassing the efficacy of fluconazole. GOs vigorous activity is attributable to sulfur compounds and phenolic monoterpenes, which as specified previously are known to disrupt membrane integrity and ergosterol biosynthesis. In contrast, EUO and RO displayed weaker or more selective inhibition; the latter demonstrated notable activity in other fungal species but had limited effects in the strains tested here.

A comparative evaluation of antioxidant, antibacterial, and antifungal activities reveals a clear structure–activity relationship among the tested essential oils. TCO, which exhibited the highest antioxidant activity (34.7 μmol TE/mL), also demonstrated pronounced antimicrobial and antifungal effects, particularly against Gram‐positive bacteria and several fungal strains. This parallel bioactivity profile is consistent with its high carvacrol content, a phenolic monoterpene known for both radical‐scavenging capacity and membrane‐disruptive antimicrobial action. GO, which ranked second in antioxidant activity (17.78 μmol TE/mL), displayed the most potent and broad‐spectrum antimicrobial and antifungal effects overall, with very low MIC values across several microorganisms. However, unlike phenolic‐rich oils, its activity is mainly attributed to sulfur‐containing compounds such as PTSO, which exert antimicrobial effects through thiol–disulfide exchange and enzyme inhibition rather than classical antioxidant pathways. In contrast, rosemary and eucalyptus oils, characterized by low antioxidant activity and a predominance of monoterpenes such as eucalyptol, showed comparatively weaker antimicrobial performance. These findings indicate that phenolic‐rich oils combine strong antioxidant and antimicrobial properties, whereas sulfur‐rich oils such as garlic can exhibit exceptional antimicrobial potency despite moderate antioxidant capacity.

### 4.4 Encapsulation Efficiency, Particle Properties, and Release Kinetics

**FIGURE 3:**
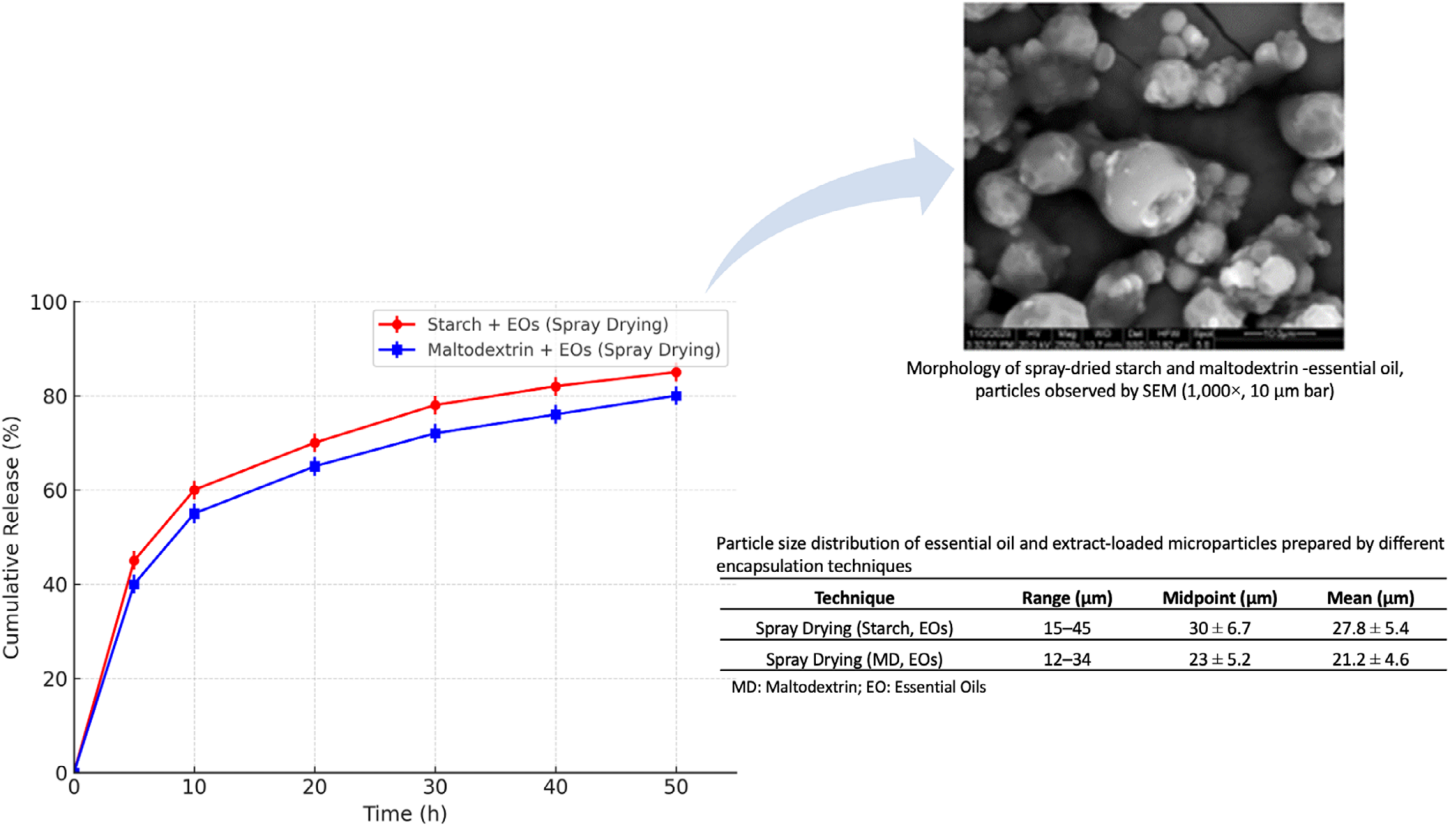
Spray‐dried starch and maltodextrin microcapsules loaded with essential oils: Cumulative release profile, morphology (SEM), and particle size distribution.

EE% varied among carrier matrices, highlighting the importance of both material choice and processing conditions. Spray drying, although widely used due to its scalability and cost‐effectiveness, yielded more moderate efficiencies (70%–77%). This trend was consistent across starch and maltodextrin matrices. The lower EE% is likely due to the high volatility and heat sensitivity of crucial oils, which can evaporate during the high‐temperature drying process. The spray‐drying formulation contained 3.85% (w/w) essential oil relative to total solids. Considering the measured encapsulation efficiencies (70%–77%), the effective EO content in the resulting microparticles was determined to range between 2.7% and 3.0% (w/w). Nonetheless, efficiencies in this range are still considered acceptable for practical applications, especially when combined with the technique's industrial feasibility. Spray drying, although slightly less efficient, remains advantageous in terms of scalability and cost, making it a valuable technique for industrial translation (Yammine et al. 2023). The particle size distribution of micro‐ and nanoencapsulated formulations was determined by laser diffraction, and morphological features were examined by SEM (Figure 3).

The encapsulation technique and carrier material had a pronounced effect on particle size and morphology. Spray drying produced particles in the range of 15–45 μm, with mean diameters ranging from 18.7 to 27.8 μm, depending on the wall material and encapsulated compound. Maltodextrin‐ and starch‐based formulations formed relatively spherical particles with smooth surfaces, as confirmed by SEM, indicating stable microcapsule formation. These sizes are typical for spray‐dried food‐grade microcapsules and are compatible with incorporation into aquaculture feeds without affecting feed texture (Choudhury et al. 2021).

The microencapsulation yield (Table 4) varied among the different EOs tested. TCO achieved a yield of 73.08%, EUO 74.49%, GO 53.44%, TO 77.81%, and RO 78.63%. Statistical analysis using Pearson's correlation revealed a strong inverse relationship between the oil content and the microencapsulation yield (*r* = −0.99). In contrast, a strong positive correlation was observed between yield and processing temperature (*r* = 0.95). These findings indicate that higher oil content tends to reduce encapsulation efficiency, whereas increased temperature improves yield, likely by enhancing the formation and stability of the encapsulating matrix.

**TABLE 4:** Encapsulation efficiency of essential oils in different carrier matrices and techniques (mean ± SD, *n* = 3).

Essential oil	Microencapsulation yield (%)	Encapsulation efficiency (%)
Rosemary oil	78.63	70.4 ± 4.3
Thyme oil	77.81	68.5 ± 3.2
Thyme conehead oil	73.08	67.2 ± 2.9
Garlic oil	53.44	71.2 ± 3.1
Eucalyptus oil	74.49	70.6 ± 4.1

The release profiles of encapsulated EOs and extracts in PBS (pH 7.4) at 25°C revealed distinct behaviors, which can be attributed to the relatively porous structure of spray‐dried particles, as well as the partial localization of bioactives at or near the particle surface during drying. While rapid release may be advantageous for immediate antimicrobial activity in aquaculture feeds, it also raises concerns about premature degradation of volatile compounds before ingestion.

While this study demonstrates the potential of EOs encapsulated by spray drying, it is limited by the use of a single encapsulation technique. Additional methods such as ionic gelation, electrospraying, or emulsification with freeze‐drying should be tested to compare efficiency, stability, and release profiles. Exploring multiple approaches would provide a more comprehensive evaluation of the most suitable strategies for aquaculture feed applications.

## 5 Conclusion

This study demonstrated that EOs from garlic, thyme, conehead thyme, rosemary, and eucalyptus exhibit distinct chemical profiles that underpin their differential bioactivities. Phenolic‐rich oils, particularly *Thymus vulgaris* and *T. capitatus* , showed superior antioxidant, antimicrobial, and antifungal activities, while garlic oil displayed strong broad‐spectrum effects due to its sulfur‐containing compounds. By contrast, eucalyptol‐dominant oils from rosemary and eucalyptus exhibited more selective and generally weaker activities.

The findings confirm that encapsulated EOs are promising natural alternatives to antibiotics in aquaculture. By combining potent bioactivity with enhanced stability and delivery, these systems can contribute to reducing antibiotic dependence and promoting sustainable fish production. Future studies should focus on in vivo validation under aquaculture conditions to translate these in vitro results into practical applications.

## Author Contributions

**Calinoiu Lavinia Florina:** conceptualization, methodology, investigation, data curation, writing – original draft, writing – review and editing, supervision. **Plamada Diana:** conceptualization, methodology, investigation, data curation, writing – original draft, writing – review and editing. **Teleky Bernadette‐Emoke:** conceptualization, writing – original draft, writing – review and editing, visualization. **Stefanescu Bianca‐Eugenia:** conceptualization, writing – original draft, writing – review and editing, visualization. **Chrysanthos Stergiopoulos:** conceptualization, methodology, visualization, writing – review and editing, writing – original draft, investigation, software. **Magdalini Krokida:** supervision, visualization, conceptualization, validation. **Ioannis Maramathas:** conceptualization, visualization, writing – original draft. **Papadaki Sofia:** methodology, investigation, data curation, writing – original draft, writing – review and editing. **Moustogianni Anna:** writing – original draft, visualization, conceptualization. **Panagiota Anagnostopoulou:** methodology, investigation, data curation. **Vodnar Dan Cristian:** conceptualization, funding acquisition, writing – original draft, validation, visualization, supervision, resources, project administration.

## Funding

This research has received funding from the European Union's Horizon Europe Framework Programme (HORIZON) under the Marie Skłodowska‐Curie grant agreement No. 101086261–FEEDACTIV. This research was supported by the Ministry of Research, Development, and Innovation and developed with the support of UEFISCDI (Project No. 41PHE, PN‐IV‐P8‐8.1‐PRE‐HE‐ORG‐2023‐0113).

## Supporting information


**Figure S1:** MIC determination against *Staphylococcus epidermidis*.
**Figure S2:** MIC determination against *Salmonella enterica*.
**Figure S3:** MIC determination against *Listeria monocytogenes*.
**Figure S4:** MIC determination against *Staphylococcus aureus*.
**Figure S5:** MIC determination against *Escherichia coli*.
**Figure S6:** MIC determination against *Pseudomonas aeruginosa*.
**Figure S7:** MIC determination against *Candida parapsilosis*.
**Figure S8:** MIC determination against *Aspergillus terreus*.
**Figure S9:** MIC determination against *Rhizopus oligosporus*.
**Figure S10:** MIC determination against *Aspergillus niger*.
**Figure S11:** MIC determination against *Aspergillus brasiliensis*.
**Figure S12:** MIC determination against *Aspergillus awamori*.
**Figure S13:** MIC determination against *Aspergillus elegans*.
**Table S1:** Time‐dependent cumulative release (%) of essential oils from spray‐dried microparticles prepared with starch and maltodextrin matrices during in vitro release experiments in PBS (pH 7.4) at 25°C. Values represent mean ± standard deviation (*n* = 3).

## Data Availability

The data that support the findings of this study are available from the corresponding author upon reasonable request.
