# Supplementary material for: Phytobiotic Essential Oils as Antibiotic Alternatives in Aquaculture: Antimicrobial and Antioxidant Properties of Garlic, Thyme, Thyme Conehead, Rosemary, and Eucalyptus
Source: Food Sci Nutr. 2026 Apr 24;14(4):e71739. doi: 10.1002/fsn3.71739 (PMC13107120; doi:10.1002/fsn3.71739)
Supplement: Supplementary file 1 — Figure S1: MIC determination against Staphylococcus epidermidis. Figure S2: MIC determination against Salmonella enterica. Figure S3: MIC determination against Listeria monocytogenes. Figure S4: MIC determination against Staphylococcus aureus. Figure S5: MIC determination against Escherichia coli. Figure S6: MIC determination against Pseudomonas aeruginosa. Figure S7: MIC determination against Candida parapsilosis. Figure S8: MIC determination against Aspergillus terreus. Figure S9: MIC determination against Rhizopus oligosporus. Figure S10: MIC determination against Aspergillus niger. Figure S11: MIC determination against Aspergillus brasiliensis. Figure S12: MIC determination against Aspergillus awamori. Figure S13: MIC determination against Aspergillus elegans. Table S1: Time‐dependent cumulative release (%) of essential oils from spray‐dried microparticles prepared with starch and maltodextrin matrices during in vitro release experiments in PBS (pH 7.4) at 25°C. Values represent mean ± standard deviation (n = 3). [file FSN3-14-e71739-s001.docx]

**Supplementary materials**

**Figure 1.** MIC determination against *Staphylococcus epidermidis*


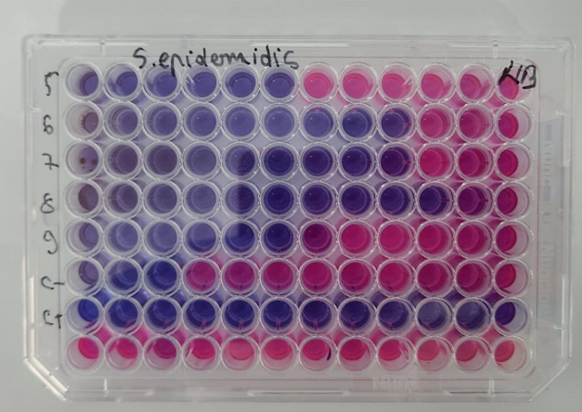


**Figure 2.** MIC determination against Salmonella enterica


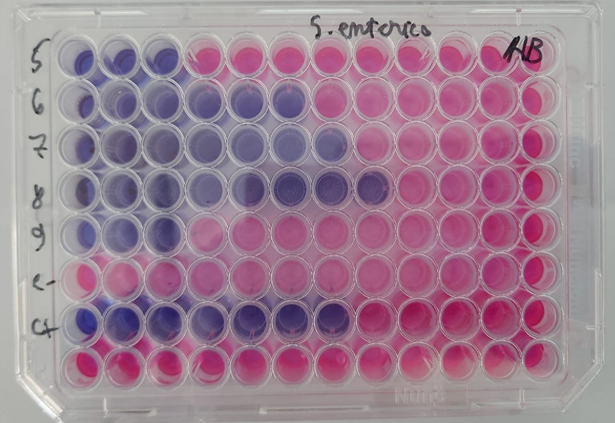


**Figure 3.** MIC determination against *Listeria monocytogenes*


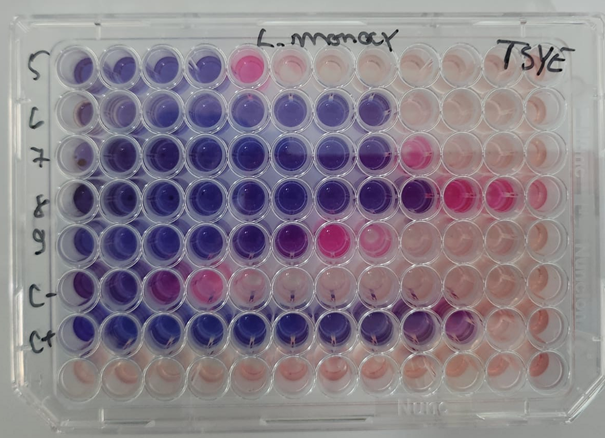


**Figure 4.** MIC determination against *Staphylococcus aureus*


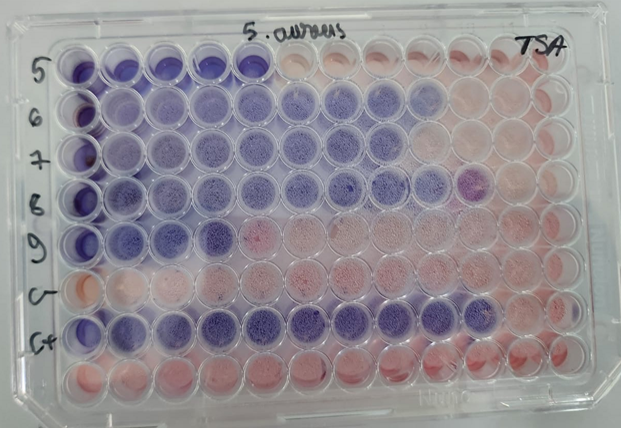


**Figure 5.** MIC determination against *Escherichia coli*


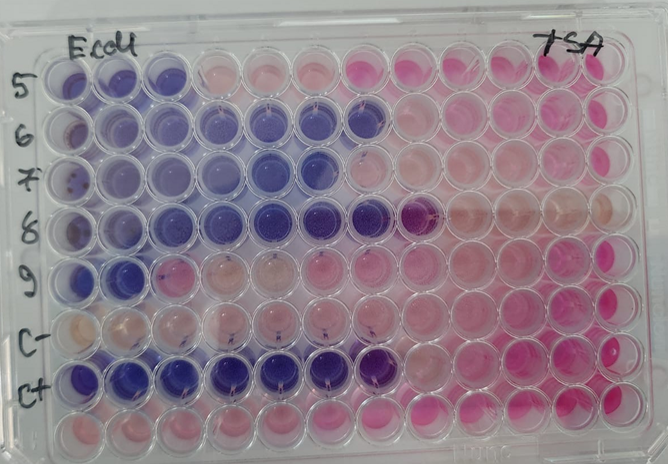


**Figure 6.** MIC determination against *Pseudomonas aeruginosa*


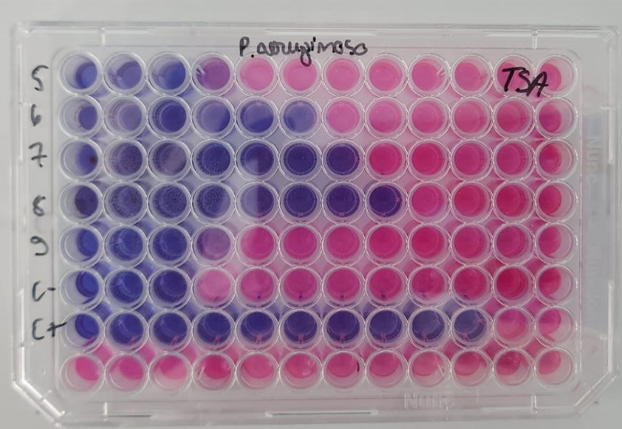


**Figure 7.** MIC determination against *Candida parapsilosis*


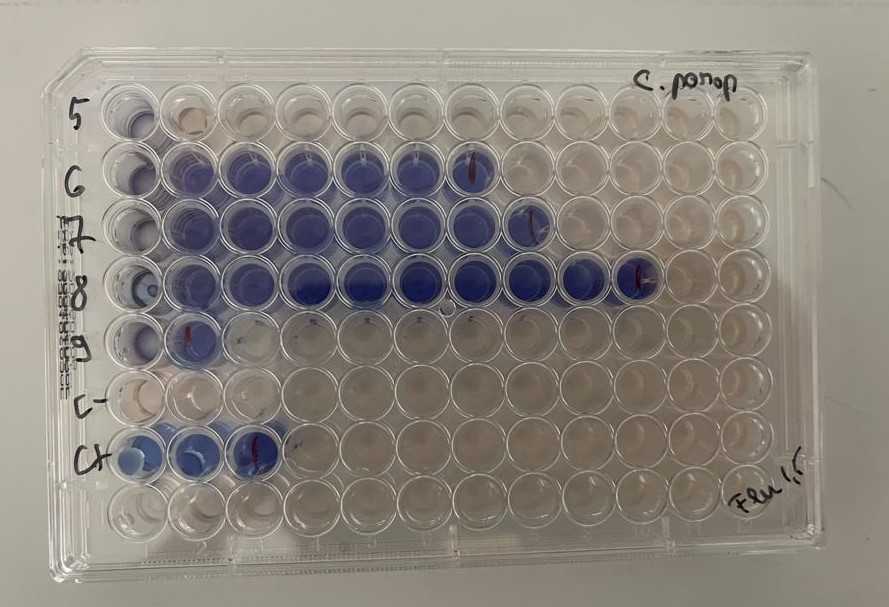


**Figure 8.** MIC determination against *Aspergillus terreus*


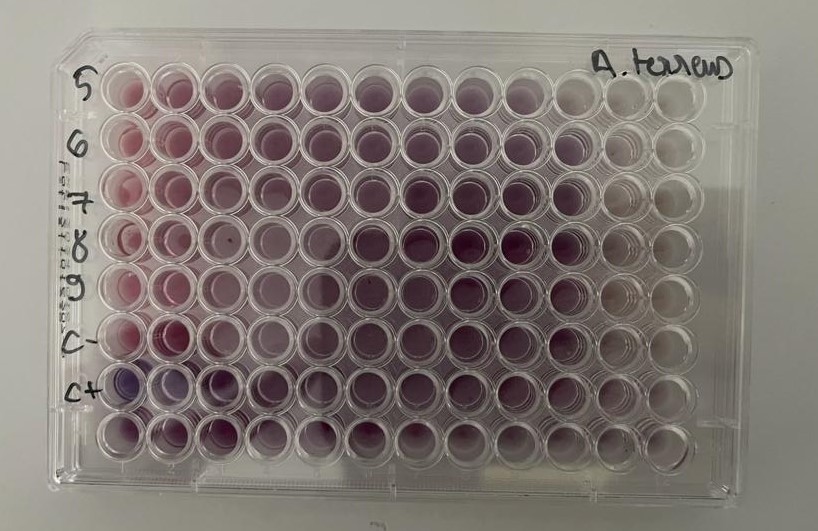


**Figure 9.** MIC determination against *Rhizopus oligosporus*


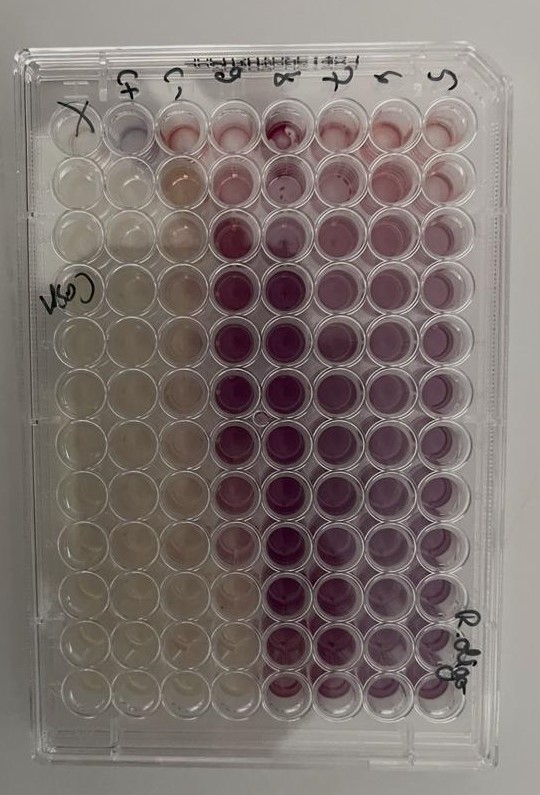


**Figure 10.** MIC determination against *Aspergillus niger*


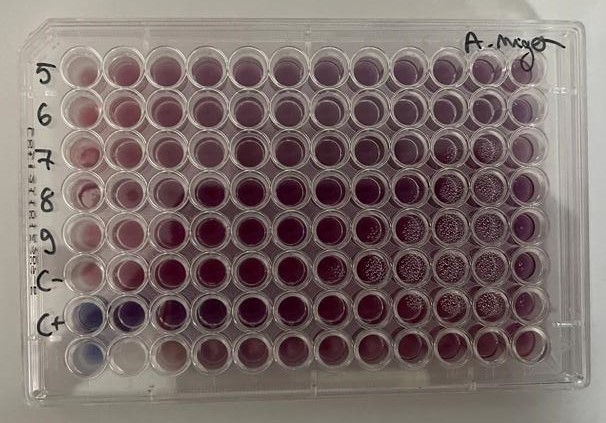


**Figure 11.** MIC determination against *Aspergillus brasiliensis*


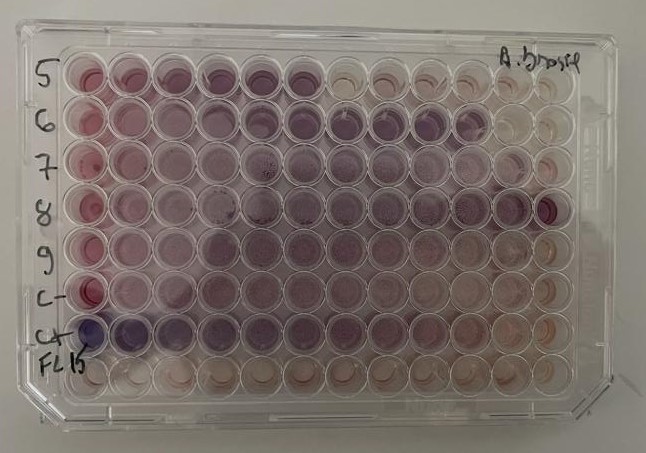


**Figure 12.** MIC determination against *Aspergillus awamori*


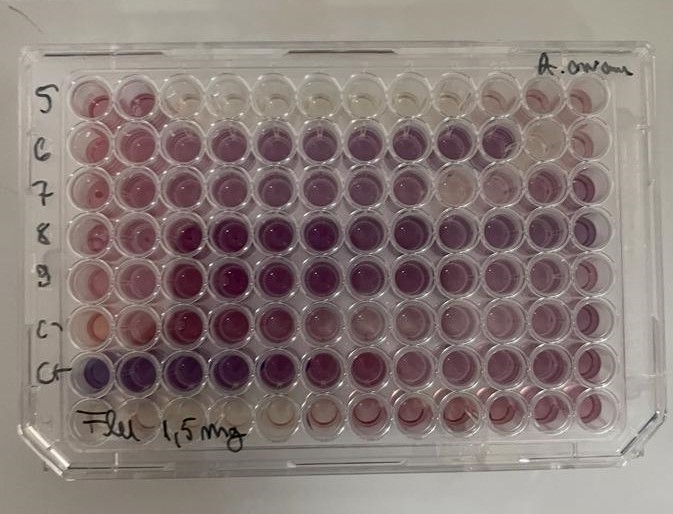


**Figure 13.** MIC determination against *Aspergillus elegans*


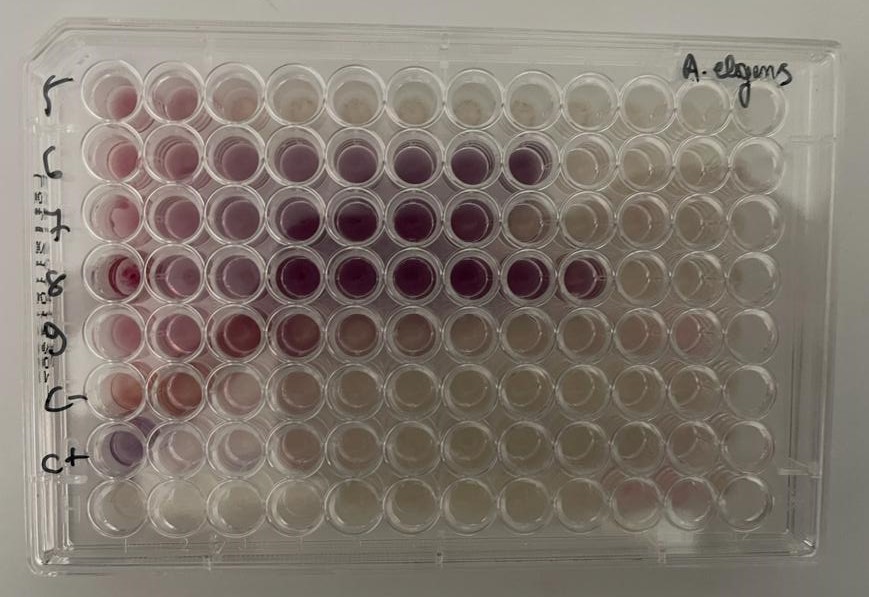


**Table S1.** Time-dependent cumulative release (%) of essential oils from spray-dried microparticles prepared with starch and maltodextrin matrices during in vitro release experiments in PBS (pH 7.4) at 25 °C. Values represent mean ± standard deviation (n = 3).

| **Time (h)** | **Starch + EO (%)** | **Maltodextrin + EO (%)** |
| --- | --- | --- |
| 0 | 0 ± 0 | 0 ± 0 |
| 5 | 45 ± 1.6 | 40 ± 1.5 |
| 10 | 60 ± 1.7 | 55 ± 1.7 |
| 20 | 70 ± 1.8 | 65 ± 1.7 |
| 30 | 78 ± 1.6 | 72 ± 1.6 |
| 40 | 82 ± 1.5 | 76 ± 1.4 |
| 50 | 85 ± 1.3 | 80 ± 1.4 |
